# Published analysis of contraceptive effectiveness of Daysy and DaysyView app is fatally flawed

**DOI:** 10.1186/s12978-018-0560-1

**Published:** 2018-06-25

**Authors:** Chelsea B. Polis

**Affiliations:** 1 Guttmacher Institute, 125 Maiden Lane, 7th Floor, New York, NY 10038 USA; 2 Department of Epidemiology, Johns Hopkins Bloomberg School of Public Health, Baltimore, MD 21205 USA

**Keywords:** Daysy, DaysyView, Fertility awareness-based methods, Contraceptive effectiveness

## Abstract

**Background:**

In March 2018, Dr. Martin C. Koch and colleagues published an analysis purporting to measure the effectiveness of the Daysy device and DaysyView app for the prevention of unintended pregnancy. Unfortunately, the analysis was flawed in multiple ways which render the estimates unreliable. Unreliable estimates of contraceptive effectiveness can endanger public health.

**Main body:**

This commentary details multiple concerns pertaining to the collection and analysis of data in Koch et al. 2018. A key concern pertains to the inappropriate exclusion of all women with fewer than 13 cycles of use from the Pearl Index calculations, which has no basis in standard effectiveness calculations. Multiple additional methodological concerns, as well as prior attempts to directly convey concerns to the manufacturer regarding marketing materials based on prior inaccurate analyses, are also discussed.

**Conclusion:**

The Koch et al. 2018 publication produced unreliable estimates of contraceptive effectiveness for the Daysy device and DaysyView app, which are likely substantially higher than the actual contraceptive effectiveness of the device and app. Those estimates are being used in marketing materials which may inappropriately inflate consumer confidence and leave consumers more vulnerable than expected to the risk of unintended pregnancy. Prior attempts to directly convey concerns to the manufacturer of this device were unsuccessful in preventing publication of subsequent inaccurate analyses. To protect public health, concerns with this analysis should be documented in the published literature, the Koch et al. 2018 analysis should be retracted, and marketing materials on contraceptive effectiveness should be subjected to appropriate oversight.

## Background

In March 2018, Martin C. Koch et al. published a paper entitled “Improving usability and pregnancy rates of a fertility monitor by an additional mobile application: results of a retrospective efficacy study of Daysy and DaysyView app” [1]. Unfortunately, this paper contains fatal flaws in the estimation of effectiveness of the Daysy device and DaysyView app for prevention of unintended pregnancy, which render the published effectiveness estimates unreliable. The published estimates are likely to be substantially higher than the actual contraceptive effectiveness of the device and app.

**Fig. 1:**
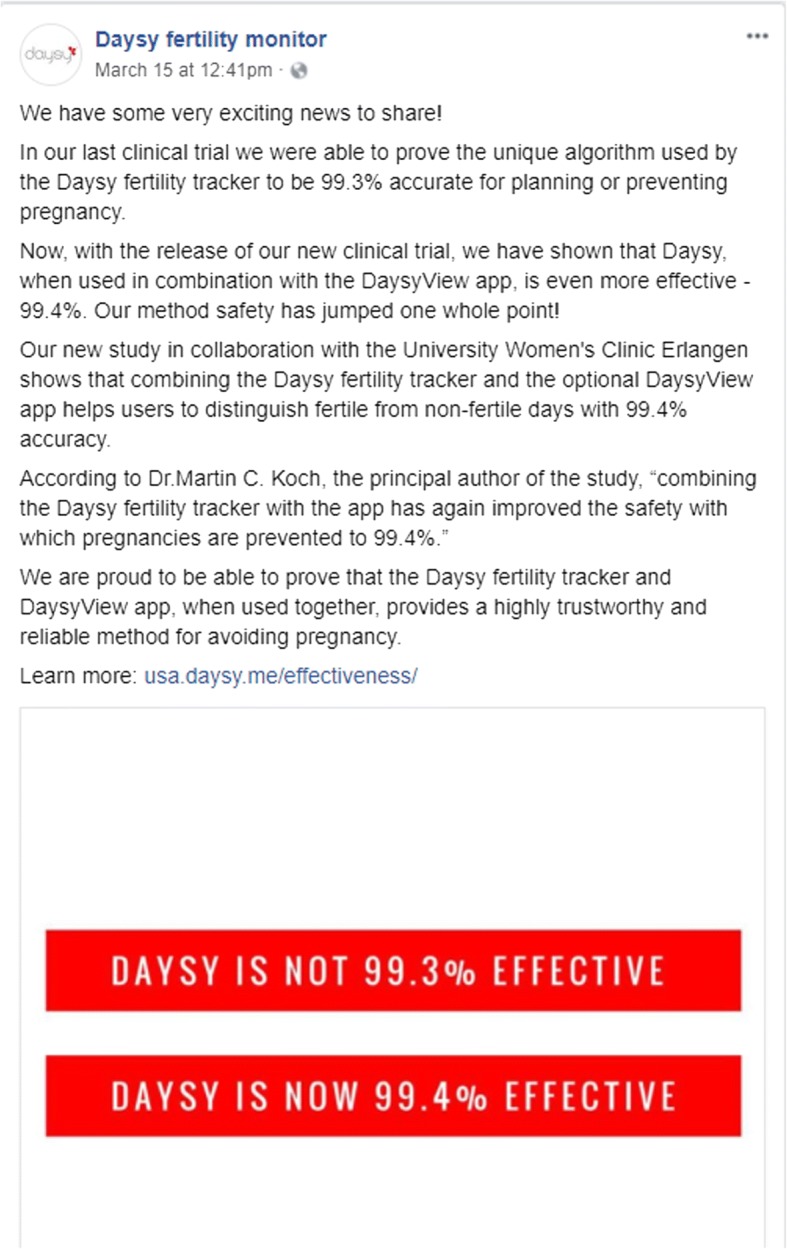
DaysyUSA Facebook post claiming a 99.4% effectiveness of Daysy and the DaysyView app (based on results of the inaccurate Koch et al. 2018 analysis)

This commentary argues that this paper merits retraction [2], given the demonstrated potential for these estimates to be used in marketing materials, leading to public confusion about the actual contraceptive effectiveness of the Daysy device and DaysyView app. The manufacturer of Daysy (Valley Electronics AG, Zurich, Switzerland) has cited the Koch et al. 2018 publication in contraceptive effectiveness claims in marketing materials (Fig. 1). This could lead to inappropriately inflated consumer confidence in the contraceptive effectiveness of Daysy and DaysyView, and could leave consumers more vulnerable to the risk of unintended pregnancy.

## Main text

The Koch et al. 2018 effectiveness estimates are fatally flawed and unreliable for multiple reasons. First, their Pearl Index calculations (which are less preferable than life table estimates [3], yet are the only estimates acknowledged in the abstract of the Koch et al. 2018 publication and the Valley Electronics marketing language shown in Fig. 1) are void of meaning since the authors inappropriately excluded from the Pearl Index calculations all women with fewer than 13 cycles of use, ignoring cycle and pregnancy information from the majority of study participants. This approach has no basis in standard effectiveness estimation approaches, and would severely underestimate unintended pregnancy rates by inappropriately excluding women who may be at greatest risk of unintended pregnancy (i.e., those who are least experienced with use of the contraceptive method and most fertile) [4]. Table 2 of Koch et al. 2018 suggests that at least 10 pregnancies occurred to women with fewer than 13 cycles (and potentially more, given broader concerns about pregnancy ascertainment, as discussed below), but these pregnancies were not included in the Pearl Index calculations.

In addition, the investigators did not prospectively collect information regarding perfect or imperfect use of the method for each cycle. Instead, they asked about overall perfect/imperfect use (intended to reflect the entire duration of use) via a retrospective survey. This precludes their ability to correctly calculate perfect use unintended pregnancy rates, in either the Pearl Index or life table calculations [5]. Thus, the so-called “perfect use” unintended pregnancy rates in Koch et al. 2018 are not comparable to correctly estimated perfect use pregnancy rates for other contraceptive methods in other studies.

Importantly, even if the inappropriate exclusion of pregnancies were corrected, the underlying data remain unreliable, given the weak approach to pregnancy ascertainment. First, the survey participation rate was low; only 13% (798/6278) of the invited population participated. Furthermore, eligibility for participation was limited to registered users with a DaysyView account, but no information is provided on what proportion of overall Daysy users this would represent. This makes the results vulnerable to selection bias and incomplete ascertainment of pregnancies among all Daysy users. Second, the authors note that if a woman indicated on the retrospective survey that she had an “unwanted” pregnancy, *then* the pregnancy was “verified from the user’s dataset” using a definition of pregnancy of “elevated temperature of longer than 18 days, or if the user stopped using the device during the luteal phase.” However, the authors do not state that temperature or device use information was evaluated among *all* women (to attempt to prospectively identify all pregnancies). If the criteria for examining the temperature/device use data were premised upon the woman self-reporting an “unwanted” pregnancy on the survey, an unknown number of pregnancies may have been excluded, given that unwanted pregnancies, including those ending in abortion, may be underreported [6, 7]. Furthermore, the survey question asked women to report “unwanted” pregnancies occurring during Daysy use. The term “unwanted” is not synonymous with “unintended” [8], and it is unclear how women may have interpreted this question – but standard contraceptive effectiveness estimates include all unintended pregnancies (including mistimed and unwanted pregnancies). Under-ascertainment of unintended pregnancies would lead to inflated estimates of contraceptive effectiveness.

Several other aspects of the questionnaire also raise concern. For example, a survey question presumably intended to ascertain if (at baseline) Daysy was being used to avoid or attempt pregnancy offered the following response options: (A) To avoid a pregnancy, (B) For family planning, (C) Both. However, response options A and B (and therefore, also C) are poorly distinguished and likely to be perceived synonymously by some respondents, meaning that even this already limited measure of baseline pregnancy intentions is unreliable. Also, this retrospective survey was unable to capture and address changing pregnancy intentions over time to attempt to prospectively characterize pregnancies as intended (and therefore, excluded from estimates of contraceptive effectiveness) or unintended (and therefore, included in estimates of contraceptive effectiveness).

While some issues in data collection and analysis are not unique to this study, they nonetheless raise additional concern about the accuracy of the estimates. For example, no inclusion/exclusion criteria were described to ensure that the analytic population was at meaningful risk of pregnancy (i.e., excluding women likely to be subfertile). Also, the majority of survey respondents (64%) reported concurrent use of contraceptive methods in addition to Daysy; the potential confounding effect of use of other methods on the effectiveness estimates is not addressed in the analysis.

For these reasons, this analysis produced estimates which cannot be understood as reflective of the true (and still unknown) contraceptive effectiveness of the Daysy device and DaysyView app. Our forthcoming systematic review of studies assessing the effectiveness of various fertility awareness-based methods (FABMs) [9] carefully considers the quality of prospective studies on effectiveness of various FABMs. Studies of extremely poor quality, such as those in Koch et al. 2018, must not be understood as providing reliable evidence on contraceptive effectiveness.

**Fig. 2:**
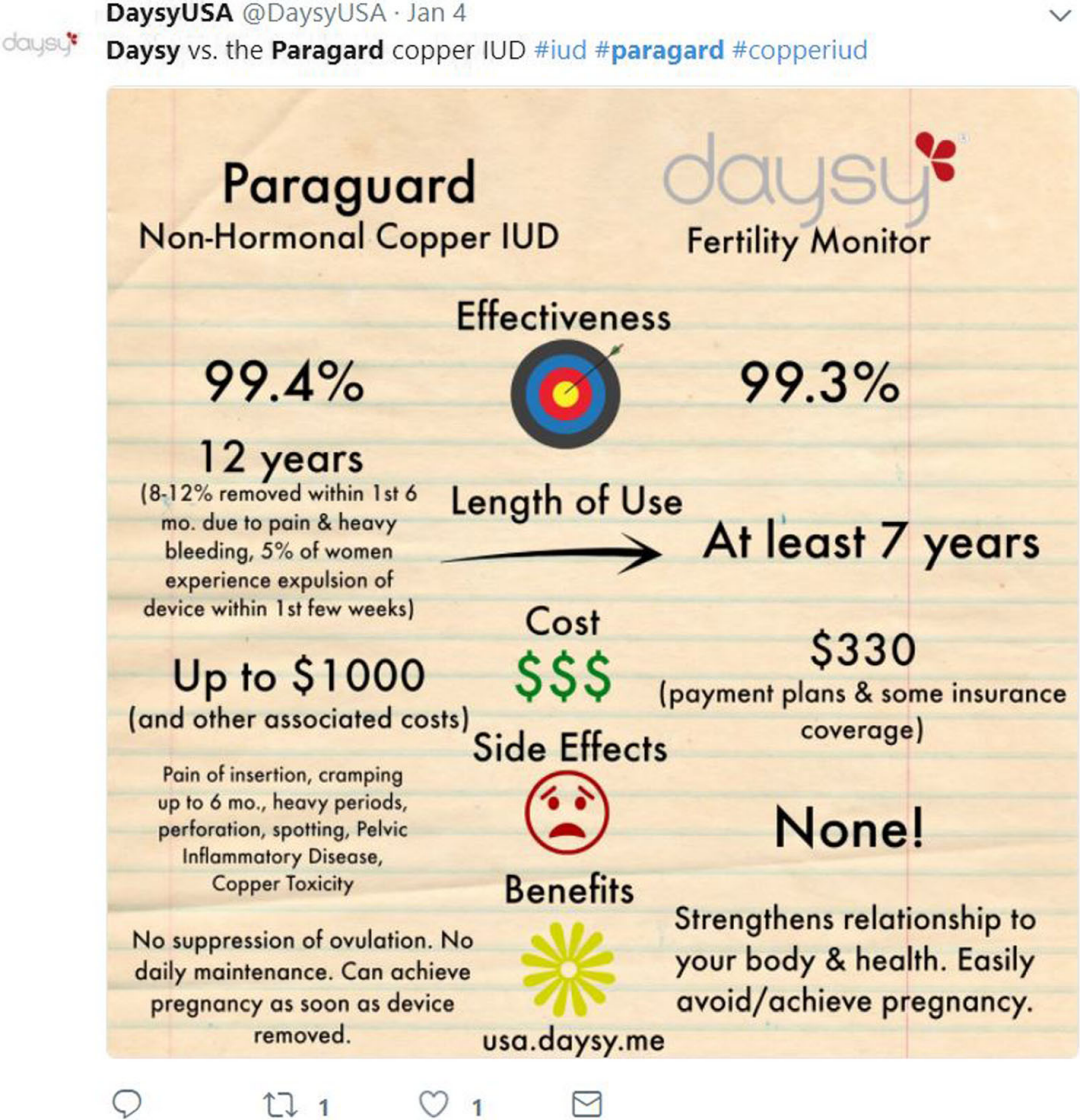
DaysyUSA Twitter post conflating robust effectiveness evidence on other contraceptive methods with misleading evidence on Daysy effectiveness, using inaccurate estimates from Freundl 1998

**Fig. 3:**
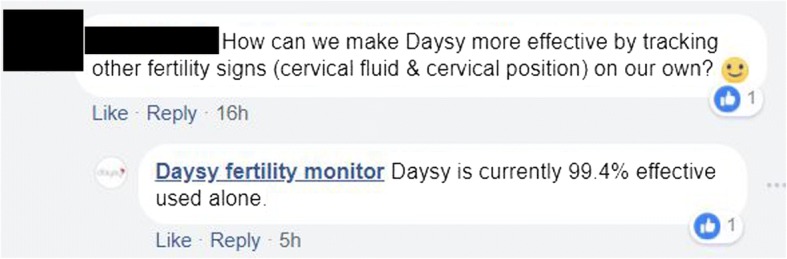
DaysyUSA on Facebook providing unreliable information to potential or existing clients about Daysy effectiveness, based on Koch et al. 2018 analyses

The manufacturer of the Daysy device, Valley Electronics, has made inaccurate marketing statements in the past (Figs. 2 and 3), based on previously published analyses [10, 11] which purported to assess contraceptive effectiveness of their products, but which also contained fatal flaws.^1^ In September 2017, I contacted the Director of Medical Affairs for Valley Electronics to directly express concerns regarding the methodological issues in these prior analyses [10, 11], and flag concern regarding the reliability of this information. I encouraged the company to “alert Daysy users and potential customers that at present, Daysy lacks sufficient evidence to be promoted as a tool for pregnancy prevention,” and to “focus instead on collecting high quality evidence to adequately assess the true effectiveness of this device” [12]. I was informed that my concerns would be discussed with the advisory board. However, the exchange appears to have been unsuccessful in encouraging the company to ensure accuracy in subsequently published estimates and marketing language. Therefore, additional steps are needed to protect scientific integrity and public health.

## Conclusions

The scientific and reproductive health communities have a responsibility to protect public health by ensuring that inaccurately conducted analyses of contraceptive effectiveness do not put unsuspecting consumers at a greater than expected risk of experiencing an unintended pregnancy. Women and couples interested in using any form of contraception, including an FABM such as Daysy, deserve robust effectiveness data on which to base their contraceptive decisions. Unfortunately, the published Koch et al. 2018 estimates are fatally flawed and inaccurate, and are likely to be substantially higher than the actual contraceptive effectiveness of the device and app. By using marketing language based on inaccurate analyses, Valley Electronics falsely increases consumer confidence in the effectiveness of the Daysy device and the DaysyView app, which could endanger the well-being of their customers. Prior efforts to directly communicate methodological concerns to the company do not appear to have led to enhanced caution in ensuring the accuracy of their published effectiveness estimates or marketing language. Therefore, to protect public health, scientific integrity, and potential consumers, these concerns should be documented in the published literature, [13] the Koch et al. 2018 analysis should be retracted, [2] and marketing materials on contraceptive effectiveness should be subjected to appropriate oversight.

## Abbreviation

FABM: Fertility awareness-based method

## Footnotes

1 For example, in addition to multiple concerns pertaining to the methods of pregnancy ascertainment in Freundl 1998 (collected via a mailed, retrospective questionnaire), the calculation which generated a 0.7 perfect use Pearl Index [10] used an incorrect denominator which included all cycles, rather than only cycles of perfect use, which would inflate perfect use effectiveness rates [3].
